# Is the hedonic hunger score associated with obesity in women? A brief communication

**DOI:** 10.1186/s13104-019-4351-8

**Published:** 2019-06-10

**Authors:** Samira Rabiei, Fatemeh Sedaghat, Reza Rastmanesh

**Affiliations:** 1 Shahid Beheshti University of Medical Sciences and Health Services, (SBMU), National Nutrition and Food Technology Research Institute, Tehran, Iran; 2 Faculty of Nutrition Sciences and Food Technology, SBMU, Tehran, Iran

**Keywords:** Hedonic hunger, Dietary patterns, Obesity

## Abstract

**Objective:**

Food intake for its hedonic properties can result in excess caloric intake. It may play a role in increasing trend of obesity in the world. Hedonic hunger may effect on dietary patterns. We assessed the association between dietary patterns and the hedonic score in obese and non-obese women. In this case–control study 140 women aged 17–44 years from an obesity club at district 4 of Tehran participated. Dietary patterns were assessed through food frequency questionnaire by factor analysis method. The hedonic score was determined using a 21-item valid questionnaire. ANOVA and Logistic regression were used to statistical analysis.

**Results:**

Factor analysis method showed that there were 2 dietary patterns named healthy and unhealthy, in order to their food items. There was not any significant trend for obesity among tertiles of healthy and unhealthy dietary patterns. The mean hedonic score was significantly higher in obese than non-obese women, (p < 0.05). The second and the third tertiles of hedonic score significantly increase the odds of obesity referring the first tertile (2.8 and 10.8, respectively). Hedonic hunger had a positive association with obesity; but there was no difference in dietary patterns of obese and non-obese women, unexpectedly.

## Introduction

Dietary patterns through energy storage are effective on body weight changes and may lead to obesity [1]. On the other hand, dietary patterns may be affected by food hedonic; since eating is pleasurable itself. By the other word, homeostatic requirements to energy is not the only stimulus for food intake, but pleasance of food may in turn stimulus the intake, regardless of to be full [2]. The activity of reward center is higher in the brain of individuals who have higher intend to eating [3] and it has been suggested that incidence of any disorder in this part of brain, increases the risk of obesity [4]. Therefore, food does not consume only for survive, but also for its pleasure and hedonic [5].

There are not enough information regarding to hedonic hunger in developing countries [6, 7]. Furthermore, the most of these studies are cross sectional, not comparative. So, to find the relation between dietary patterns and hedonic hunger with obesity, the current study has compared major dietary patterns and hedonic hunger in obese and non- obese women for the first time in Iran. The aim of this study was to compare the hedonic hunger scores in obese and non-obese women and its relation to their dietary patterns.

## Main text

### Methods

This case control study was conducted on 140 women aged 17–44 referring to fitness club in district 4 in Tehran, by easy sampling method. We used OR = 3 to achieve the highest power and the lowest error. It means that the probable of adherence to an unhealthy dietary pattern in obese individuals is 3 times more than non-obese individuals. Therefore, 46 obese women entered in case group and 92 normal weight women entered in control group. We excluded pregnant, lactating and menopause women, patients with any endocrine disorders, liver diseases, renal disease and women who had adherence to any kind of weight loss therapy during last year. We also excluded consumers of endocrine function effective drugs, oral contraceptive drugs, weight loss drugs, anti-depression drugs and alcohol. Weight and height were measured and body mass index (BMI) was calculated through dividing the weight (kg) by the squared height (m^2^). We considered women who had BMI equal to 30–45 kg/m^2^ as obese and BMI equal to 18.5–25 kg/m^2^ as normal weight. The semi quantitative food frequency questionnaire with 168 food items that was validated by Esmaeil zadeh [8] and Mirmiran [9] to determine dietary patterns, was used to access to dietary intakes. Principal component analysis (PCA) method (also named factor analysis) was used to determine dietary patterns.

To access hedonic scores, participants completed the 21-item hedonic hunger questionnaire [10]. The validity of this questionnaire was confirmed with correlation coefficient equivalent to 0.78–0.89. The higher score of this questionnaire, the higher tendency to food hedonic intake. General information were gathered by a self-reported questionnaire. Spss.17 was used to statistical analysis. Independent T-test and Mann whitney U test were used to compare quantitative data with normal and abnormal distribution, respectively. K-square test was used for qualitative data. To compare distribution of hedonic scores among dietary patterns tertiles, ANOVA test was used and to pairwise comparison, Tukey’s HSD was used if there was any significancy. Logistic regression was used to determine the relation of hedonic hunger and obesity after adjusting the confounders and to determine odds ratio of obesity for dietary patterns and hedonic scores with 95% confidence interval.

### Results

The mean of age, calorie intake and physical activity was 30.7 years, 2367.2 kcal, 36.3 MET/h/day in case group and 30.6, 2341, 37.5 in control group, respectively. These variables did not shown any significant differences between both groups. There were not any significant difference in smoking status and dietary supplements consumption between two groups (Data are not shown).

Two major Dietary patterns were determined by PCA method that was named healthy and unhealthy because of their contents. The healthy dietary pattern included: fruits, legumes, vegetables, olive, nuts, egg, dry seeds, whole grains, fish, potato, poultry and sour. The unhealthy diet included French fries, mayonnaise, synthetic fruit juices, high fat dairy, processed meat, snacks, cookies, desserts, coke, refined grains, red meat, organ meats and oils. These two patterns, overall, can explain 17.5% of total variance of dietary patterns.

The mean of total score of hedonic hunger questionnaire in cases and controls was 63.8 and 48.8, respectively, which was significantly different between two groups (p < 0.05).

Odds ratio of obesity did not have any significant difference among tertiles of healthy and unhealthy patterns.

As shown in Table 1, p value for trend is significant for OR of obesity among tertiles of hedonic scores. The third tertile of hedonic score increased OR for obesity comparing to the first tertile.

**Table 1:** Odds ratio for obesity in tertiles of hedonic score

Hedonic score	OR
Tertiles of hedonic scores	
The first tertile	1
The second tertile	2.1 (0.7–6)
The third tertile	7.4 (2.7–20)
P trend	< 0.05

As Table 2 shown, distribution of hedonic scores did not have any significant trend among tertiles of healthy pattern, although, it was significant in the third tertile comparing to the second tertile of unhealthy pattern (p < 0.05).

**Table 2:** Distribution of hedonic scores in tertiles of dietary patterns

	Dietary pattern tertiles	Hedonic score
Healthy dietary pattern	The first tertile	51.7 ± 15
	The second tertile	54.8 ± 17.1
	The third tertile	54.8 ± 20.1
P trend		0.6
Unhealthy dietary pattern	The first tertile	54.4 ± 17.3
	The second tertile	48.4 ± 14.3
	The third tertile	58.519.2^a^
P trend		< 0.05

^a^Significant difference comparing with the second tertile

### Discussion

This study showed that there is not any association between dietary patterns and obesity. To now, several studies assessed the association of dietary patterns and obesity. For example, in cohort study of Tehran lipid and Glucose Study (TLGS), 5 dietary patterns were determined that of them, traditional pattern and western diet were in positive relation to obesity [11]. We found that vegetables and egg in the healthy diet, while in their study, these food item were in traditional diet. Carrera et al. also found no association between dietary patterns and obesity, same as our study [12]. Our findings were also in agree with Naja, et al. that did not find any association between obesity and traditional diet which was similar to our healthy diet [13]. Achieving non conclusive results regard to dietary patterns and obesity may be due to differences in the nature of dietary patterns. Different design of study and food questionnaires may be of other reason for this controversies. Furthermore, complexity nature of traditional diet may lead to difficult interpretation of results.

In our study, the hedonic score in obese women was significantly higher than non-obese women. This finding is according to some of the other studies [4, 14, 15]. We also found that hedonic scores in the highest tertile of unhealthy dietary pattern is significantly higher than the lowest tertile of this pattern. However, there was not any significant trend among tertiles of healthy dietary pattern regard to hedonic score. We may conclude that hedonic hunger in obese women lead to unhealthy food choices and finally to unhealthy dietary intakes. It can develop obesity and other its risk factors. In the other words, obese women enjoy eating, more than non-obese women. Therefore, identification of unhealthy food choices which consume through hedonic mechanisms by obese people, is very important, because it can be beneficial in both hedonic obesity prevention and treatment through designing drugs to suppress rewards pathways relating to food intake.

Lack of significant difference between dietary patterns of obese and non-obese people and considering that none of our participants did not have any metabolic disorders to be effective on weight, suggested that some of the other factors, other than nutrition, physical activity and metabolic factors may play role in weight gain.

## Limitation

We conducted this study on women in district 4 of Tehran. So, we could not generalize our results to population of Tehran. We recommended to conduct a study on both sexes and in some different districts in Tehran, so that to be representative population of Tehran.

## Availability of data and materials

The raw data gathered for the current study cannot be publicly available because of the participants’ privacy and our institute regulations, as approved by the research ethics committee and has been written in consent form. All data were delivered and archived in the National Nutrition and Food Technology Research Institute of Shahid Beheshti University of Medical Sciences.
